# Supplementary material for: Nash equilibria in human sensorimotor interactions explained by Q-learning with intrinsic costs
Source: Sci Rep. 2021 Oct 21;11:20779. doi: 10.1038/s41598-021-99428-0 (PMC8531365; doi:10.1038/s41598-021-99428-0)
Supplement: Supplementary file 1 — Supplementary Information. [file 41598_2021_99428_MOESM1_ESM.pdf]

Nash equilibria in human sensorimotor interactions explained by Q-Learning with intrinstic costs

Cecilia Lindig-León [*,1,†], Gerrit Schmid [1,†],Daniel A. Braun [1]

[1]Institute of Neural Information Processing, Faculty of Engineering, Computer Science and Psychology, Ulm University, Germany.

[†] These authors contributed equally to this work.

Cecilia Lindig-León, cecilia.lindig-leon@uni-ulm.de

Nash equilibrium solutions in the continuous matching pennies games

Let the actions of player 1 and player 2 be two independent random variables X_1 and X_2 on $\Omega = [0, 1]$ and let $M \in \mathbb{R}^{2\times 2}$ be the payoff matrix for the continuous (asymmetric) matching pennies game, i.e.

$$(M_{i,j,1}) = \begin{pmatrix} a & 0 \\ 0 & 1 \end{pmatrix}$$
$$(M_{i,j,2}) = \begin{pmatrix} 0 & 1 \\ 1 & 0 \end{pmatrix}$$

where a is a constant. Then every pair of proper probability distributions of X_1 and X_2 such that $\mathbb{E}[X_1] = \frac{1}{2}$ and $\mathbb{E}[X_2] = \frac{1}{a+1}$ is a Nash equilibrium.

Proof. The continuous payoff U_k for player k is given by the payoff interpolation

$$U_k(x_1, x_2) = (1 - x_1)\left(x_2 M_{1,1,k} + (1 - x_2)M_{1,2,k}\right) + x_1\left(x_2 M_{2,1,k} + (1 - x_2)M_{2,2,k}\right),$$

Then the expected payoff of player k is

$$\mathbb{E}[U_k] = \int_{\Omega^2} p(X_1 = x_1, X_2 = x_2)U_k(x_1, x_2)dx,$$

where p is the joint distribution of X_1 and X_2. Since X_1 and X_2 are assumed to be independent p factorizes into $p(X_1 = x_1, X_2 = x_2) = p_1(x_1)p_2(x_2)$ and it follows that

$$\begin{aligned}
\mathbb{E}[U_1] &= \int_\Omega \int_\Omega p_1(x_1)p_2(x_2)\left((1 - x_1)(x_2 a) + x_1(1 - x_2)\right) dx_2\, dx_1 \\
&= \int_\Omega p_1(x_1)\left(\int_\Omega p_2(x_2)x_1\, dx_2 + \int_\Omega p_2(x_2)x_2 a\, dx_2 - \int_\Omega p_2(x_2)(1 + a)x_1 x_2\, dx_2\right) dx_1 \\
&= \int_\Omega p_1(x_1)\left(x_1 + a\mathbb{E}[X_2] - (1 + a)x_1\mathbb{E}[X_2]\right) dx_1 \\
&= a\mathbb{E}[X_2] + \int_\Omega p_1(x_1)(1 - (1 + a)\mathbb{E}[X_2])x_1\, dx_1,
\end{aligned}$$

which is independent of $p_1(x_1)$ whenever $\mathbb{E}[X_2] = \frac{1}{1+a}$. In that case player 1 is indifferent about the distribution of his actions, making it a Nash-strategy for player 2.

Similarly, it is

$$\begin{aligned}
\mathbb{E}[U_2] &= \int_\Omega \int_\Omega p_1(x_1)p_2(x_2)\left((1-x_1)(1-x_2)+x_1x_2\right)\,dx_2\,dx_1 \\
&= \int_\Omega p_2(x_2)\left(\int_\Omega p_1(x_1)\,dx_1 - \int_\Omega p_1(x_1)x_1\,dx_1 - \int_\Omega p_1(x_1)x_2\,dx + 2\int_\Omega p(x_1)x_1x_2\,dx_1\right)\,dx_2 \\
&= (1-\mathbb{E}[X_1]) + \int_\Omega p_2(x_2)(2\mathbb{E}[X_1]-1)x_2\,dx_2,
\end{aligned}$$

which is independent of $p_2(x_2)$ whenever $\mathbb{E}[X_1] = \frac{1}{2}$. □

Categorical Analysis

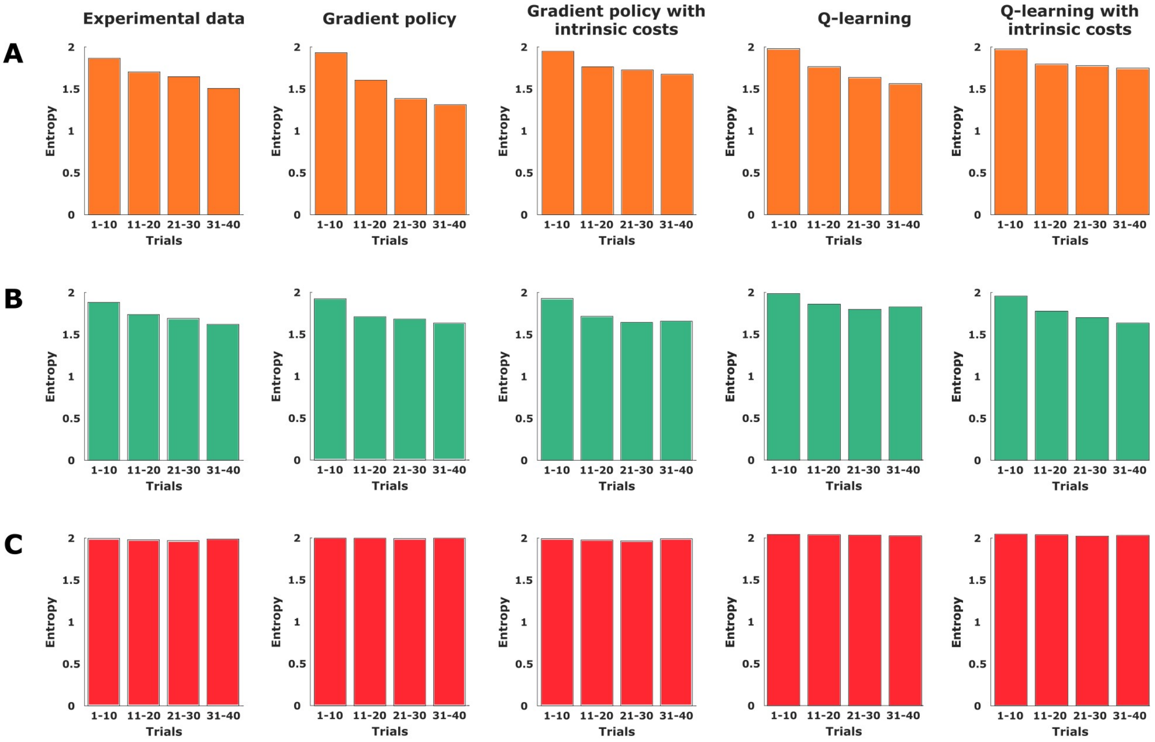

Figure 1: Entropy of the joint action distribution. The joint entropy is computed over the categorized experimental data and the binary models on blocks of 10 trials each, for A) prisoners' dilemma, B) asymmetric matching pennies, and C) symmetric matching pennies. Created using MATLAB R2021a (https://www.mathworks.com).

Continuous Analysis

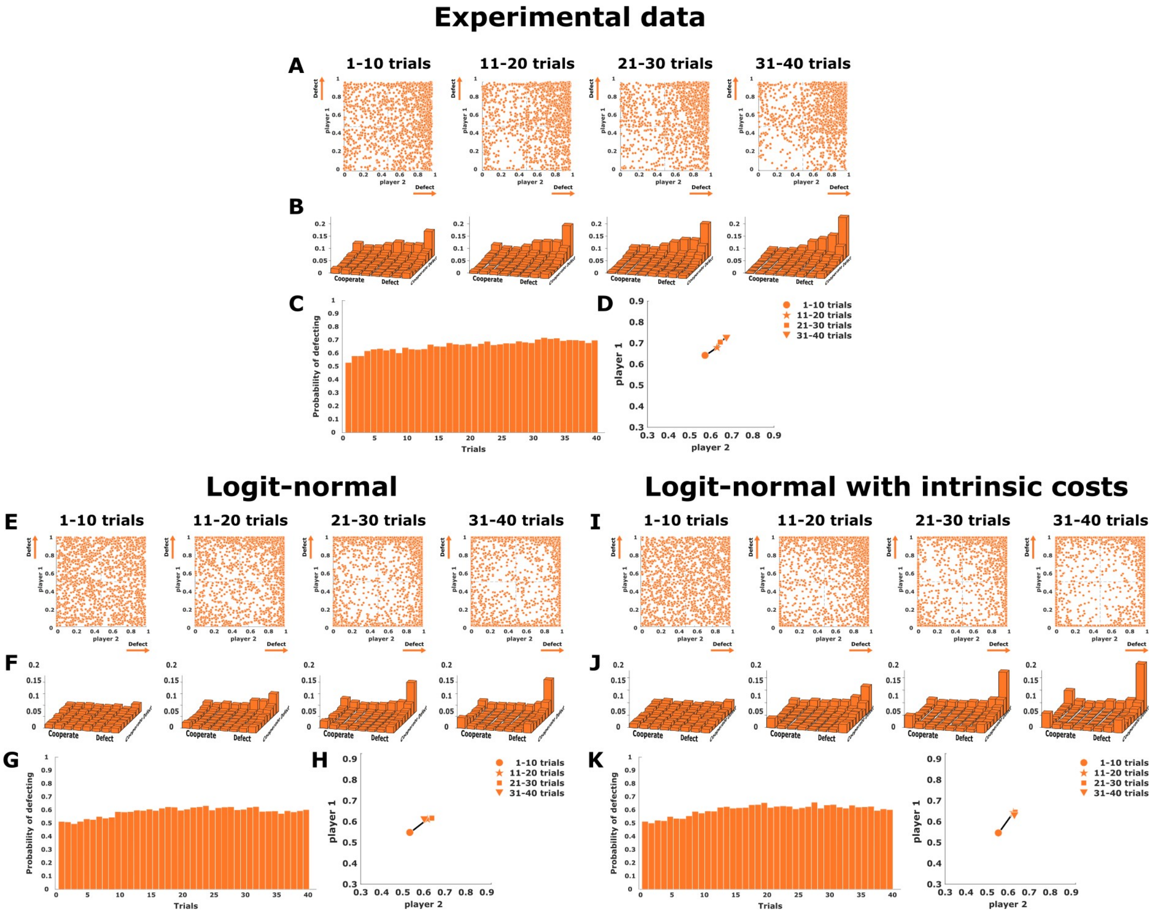

Figure 2: Prisoner's dilemma, logit normal. (A), (E), and (I) show scatter plots of final decisions in the x_1x_2-plane, where subjects' actions are expected to cluster around the single pure Nash equilibrium located in the top-right corner at position (1,1). (B), (F), and (J) show two-dimensional histograms binning the experimental scatter plots. (C), (G), and (K) represent the change of the mean endpoints (averaged for both players) for each trial across the block of 40 trials. (D), (H), and (L) show the direction of adaptation in the endpoint space. The experimental data is shown at the top, the two continuous models are shown below. Created using MATLAB R2021a (https://www.mathworks.com).

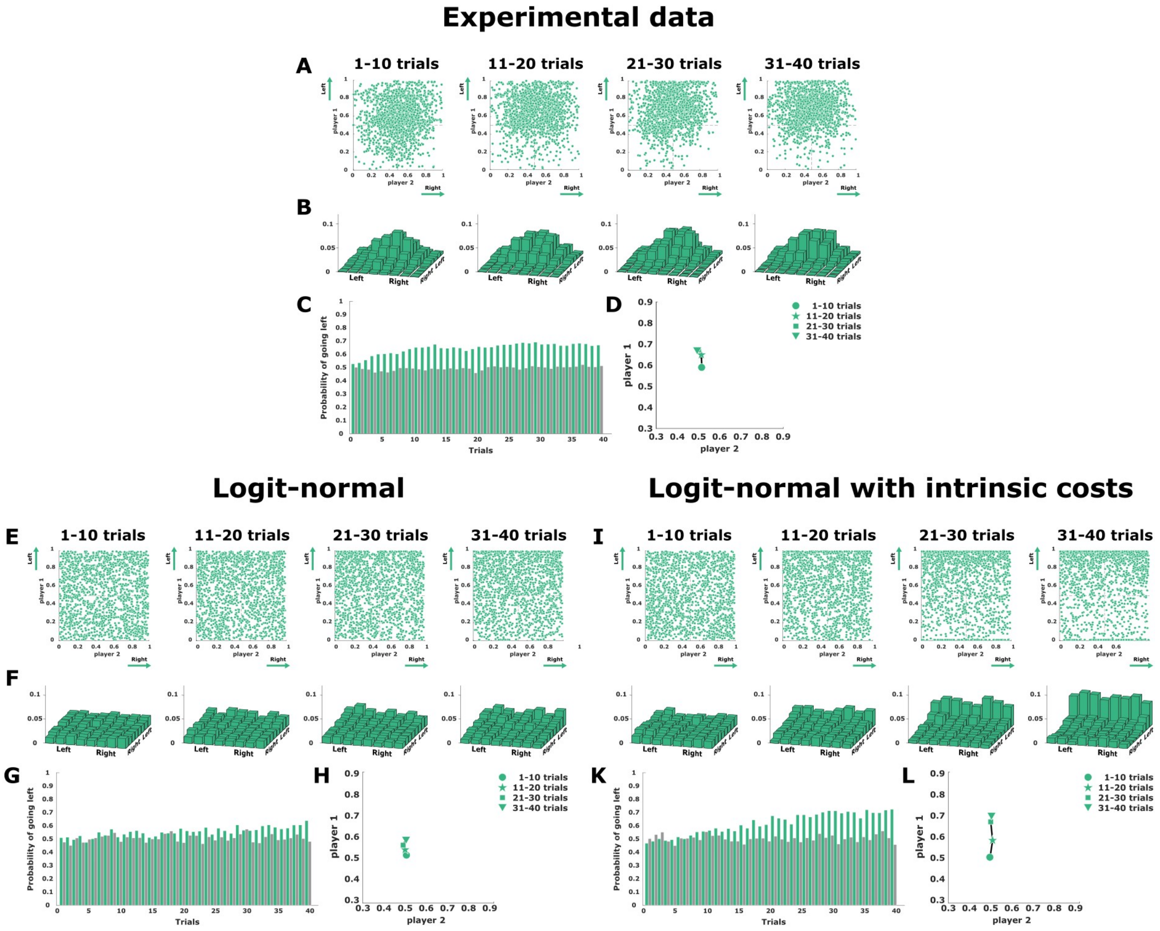

Figure 3: Asymmetric matching pennies, logit normal. (A), (E), and (I) show final decisions as a scatter plot in the x_1x_2-plane, where subjects' actions are expected to cluster in top quadrants along each mini-block of 10 trials. (B), (F), and (J) show a two-dimensional histogram binning of the experimental scatter plots. (C), (G), and (K) present the change over the mean endpoint (averaged for both players) for each trial across the block of 40 trials. (D), (H), and (L) show the direction of adaptation in the endpoint space. The experimental data is shown on the top, the two continuous models are below. Created using MATLAB R2021a (https://www.mathworks.com).

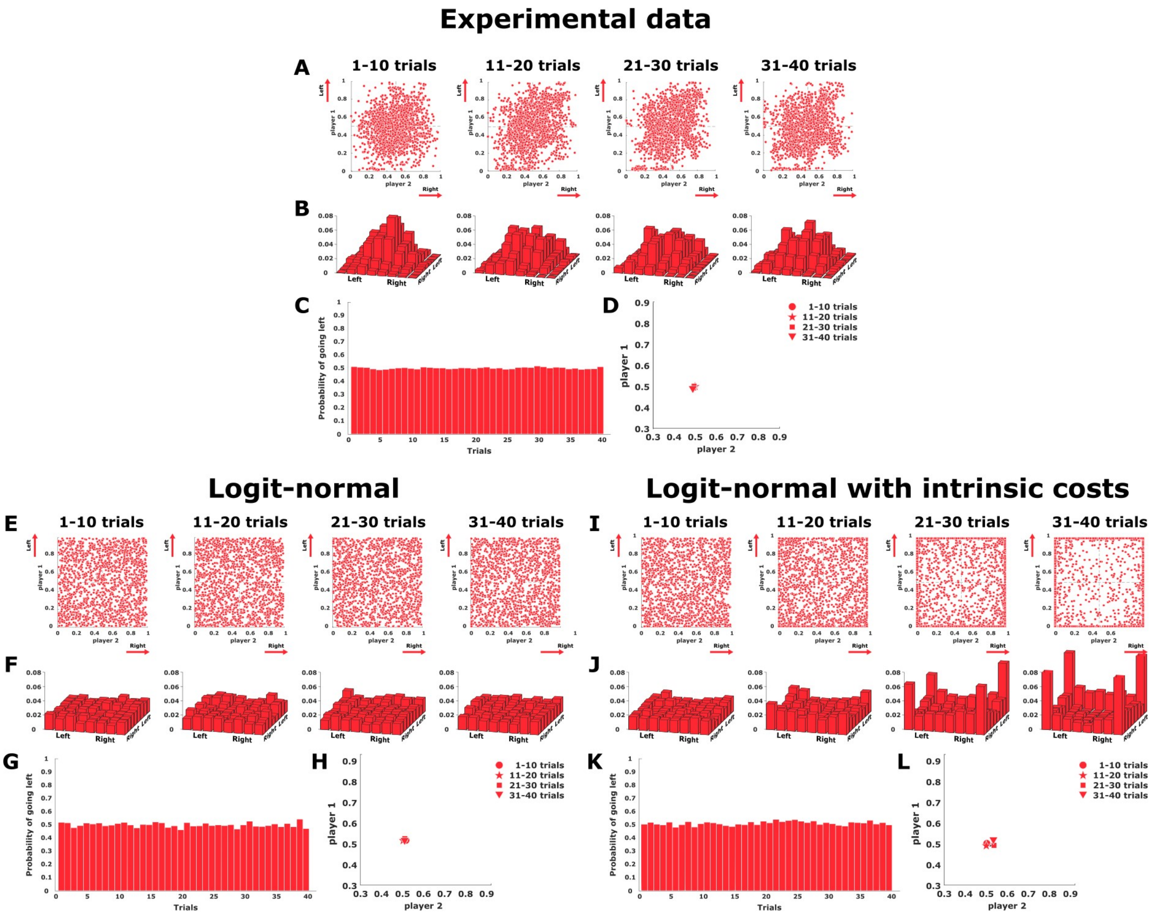

Figure 4: Symmetric matching pennies, logit normal. (A), (E), and (I) show final decisions as a scatter plot in the x_1x_2-plane, where subjects' actions are expected to cluster around the center of the workspace along each mini-block of 10 trials. (B), (F), and (J) show a histogram binning of the experimental scatter plots. (C), (G), and (K) present the change over the mean endpoint (averaged for both players) for each trial across the block of 40 trials. (D), (H), and (L) show the direction of adaptation in the endpoint space. The experimental data is shown in the top, the two continuous models are below.Created using MATLAB R2021a (https://www.mathworks.com).

Game	Euclidean distance				Mean-squared error			
	PG	PG_{IC}	QL	QL_{IC}	PG	PG_{IC}	QL	QL_{IC}
Prisoners' dilemma	0.14	0.11	0.14	0.08	0.011	0.005	0.014	0.001
Asymmetric MP	0.13	0.15	0.12	0.09	0.002	0.005	0.001	0.001
Symmetric MP	0.13	0.13	0.08	0.08	0.0003	0.0003	0.0002	0.0001

Table 1: Models evaluation. Euclidean distance and mean-squared error between the two-dimensional histograms of the subjects' data and the simulated histograms for all games and learning algorithms (PG: Policy Gradient, QL: Q-learning, IC: Intrinsic Costs).
